# 

**Published:** 1860-09

**Authors:** 


### CONTENTS.

ORIGINAL ESSAYS AND COMMUNICATIONS.

Bucal Secretions. By J. Taft........ 121
Sensitive Dentine. By Geo. F. Foote, M. D........ 129
Artificial Dentures. By Dr. J. Allen........ 134
Mallet-ing........ 142

PROCEEDINGS OF SOCIETIES.

American Dental Convention........ 143

Selections........ 180

Editorial........ 181

